# Integration of Low-Cost GNSS and Monocular Cameras for Simultaneous Localization and Mapping

**DOI:** 10.3390/s18072193

**Published:** 2018-07-07

**Authors:** Xiao Chen, Weidong Hu, Lefeng Zhang, Zhiguang Shi, Maisi Li

**Affiliations:** 1 Science and Technology on Automatic Target Recognition Laboratory, National University of Defense Technology, Changsha 410073, China; wdhu@nudt.edu.cn (W.H.); zlfeng1110@126.com (L.Z.); szg0428@tom.com (Z.S.); 2 Electrical and Computer Engineering Department, Carnegie Mellon University, 5000 Forbes Ave, Pittsburgh, PA 15213, USA; maisili@andrew.cmu.edu

**Keywords:** SLAM, GNSS, low-cost, sensor fusion, navigation, ORB-SLAM, bundle adjustment

## Abstract

Low-cost Global Navigation Satellite System (GNSS) receivers and monocular cameras are widely used in daily activities. The complementary nature of these two devices is ideal for outdoor navigation. In this paper, we investigate the integration of GNSS and monocular camera measurements in a simultaneous localization and mapping system. The proposed system first aligns the coordinates between two sensors. Subsequently, the measurements are fused by an optimization-based scheme. Our system can function in real-time and obtain the absolute position, scale, and attitude of the vehicle. It achieves a high accuracy without a preset map and also has the capability to work with a preset map. The system can easily be extended to create other forms of maps or for other types of cameras. Experimental results on a popular public dataset are presented to validate the performance of the proposed system.

## 1. Introduction

Localization of a device in an unknown environment is critical in many fields. The Global Navigation Satellite System (GNSS) receivers and visual simultaneous localization and mapping (visual-SLAM) systems are popular navigation solutions.

In the simplest SLAM systems, a monocular camera is used to determine the ego-motion and build a map. This system does not rely on any external equipment and can work effectively in GNSS-denied environments. With recent SLAM approaches [1,2,3], very high localization and mapping accuracy can be obtained for a medium-length trajectory. Therefore, SLAM systems are often used in autonomous navigation [4], path planning [5], environment reconstruction [6], scene understanding [7], and so on. However, SLAM systems accumulate error while running, which grows almost linearly with running distance or time [8]. Another drawback of SLAM is that the absolute pose of the device cannot be observed. It only measures the relative pose with repect to the first frame. Moreover, if a monocular camera is used, the absolute scale of the system is ambiguous [9].

In the case of outdoor navigation, a GNSS receiver is currently the most commonly used system. These provide localization measurements with an accuracy of <5 m in open-sky areas. However, in applications such as autonomous driving and unmanned aerial vehicle control, such positioning accuracy does not meet practical requirements [10]. Although RTK(Real-time kinematic)-GNSS can achieve a positioning accuracy of centimeters or even millimeters, it requires cooperative ground station assistance and is very expensive. Therefore, RTK-GNSS is not commonly used. Moreover, GNSS receivers can only measure the position of the device; obtaining the attitude is not easy. To determine the attitude, an inertial measurement unit (IMU) is often used. However, Agrawal, et al. [11] points out that the estimation error of low-cost GNSS and IMU integrated systems is still high. Navigation systems based on GNSS or GNSS–IMU require a tradeoff between price and accuracy. In addition, GNSS receivers will always be affected by environmental factors. For example, in tunnels or underground, the system cannot receive navigation signals, and in buildings or valleys, signals are shadowed or suffer from multipath effects. Even in GNSS–IMU coupled systems, the IMU prediction error grows quadratically when GNSS data become invalid, which means that the coupled system cannot run for a long time after losing the GNSS signal.

The complementary nature of visual-SLAM and GNSS results in numerous benefits. The integration of the two systems greatly improves performance. Dusha et al. [12] proved that visual-GNSS integrated navigation systems can observe absolute pose. GNSS receiver observations can be used to provide the absolute pose and scale for SLAM systems and limit drift. Additionally, with visual measurements, a GNSS receiver is capable of attitude estimation, and the localization error is smoothed. Moreover, the camera and the GNSS receiver can be very cheap and portable. Therefore, the integration of GNSS and SLAM systems has received extensive attention from researchers [13,14,15].

In this study, we chose ORB-SLAM [1] as the visual module and propose a data fusion scheme to integrate the visual and GNSS information. ORB-SLAM [1] is a state-of-the-art SLAM system that uses ORB [16] features for tracking, mapping, loop closure, and relocalization. The system utilizes monocular, stereo, or RGB-D cameras, runs in real-time, and provides high localization accuracy. Regarding data fusion, at present, most visual-GNSS integrated navigation systems fuse data by filtering-based methods. However, in modern SLAM systems, we found that an optimization-based method has been proven to have higher accuracy and computational efficiency. When using a low-cost GNSS, the measurements are noisy. Hence, we introduce an optimization-based data fusion method to improve the navigating performance. We first studied the data alignment method of the two devices. After that, the information fusion framework was designed. Compared with the original ORB-SLAM system, we propose big bundle adjustment (big BA) and global BA threads to utilize GNSS information and maintain global consistency in localization and mapping. We name this system GVORB (GNSS-Visual-ORB-SLAM).

The paper is organized as follows: We review related work on SLAM and visual-GNSS coupled navigation in Section 2. Then, the proposed system is introduced in Section 3 and Section 4. In Section 5, we evaluate the performance of our algorithm through experiments on the KITTI [17] dataset. In Section 6, the application and extensibility of the system are discussed. Finally, we provide a summary in Section 7.

## 2. Related Work

### 2.1. Visual SLAM

The concept of SLAM was firstly proposed in 1986 [18]. Initially, the filtering-based backend was used to process visual measurements. For example, Montemerlo et al. [19] proposed FastSLAM, in which a Rao–Blackwellized particle filter was used to estimate the pose of the camera. In 2003, Davison et al. proposed MonoSLAM [20], in which an extended Kalman filter (EKF) was used to filter the camera pose and position of map points (MPs). MonoSLAM was the first real-time and open-source SLAM system and greatly promoted the development of SLAM research. After that, Klein et al. presented PTAM [21]. It uses an optimization- and keyframe-based approach with two parallel processing threads. The optimization algorithm in PTAM is called BA, which was first used in structure from motion (SfM). BA optimizes camera poses and MP positions jointly to minimize the MP reprojection error. Benefitting from the sparsity of the SLAM problem, BA can provide results efficiently, and the estimation accuracy of BA is higher than that of a filtering-based estimator [22]. Additionally, keyframes in PTAM are used to efficiently estimate camera poses and reduce redundancy of information. These characteristics led to the development of modern SLAM. Nowadays, mainstream SLAM methods, such as ORB-SLAM [1], DSO [2], and LSD [23], are based on keyframe- and optimization-based algorithms.

The visual information processing in SLAM can be divided into direct and indirect methods. DSO [2] is based on the direct method, which uses the pixel’s grayscale variation directly to determine the pose, with which more visual information can be obtained and runtime can be very fast. However, because of the large amount of uncertainty in data alignment, the method is more likely to fall into local extrema. Indirect methods, such as ORB-SLAM [1], first extract features from the input images and match them with features in the map. By minimizing the reprojection error, the pose can be determined. The advantage of the indirect method is that it is more robust during strong motion. Moreover, it is easier to deal with relocalization and loop closure. Therefore, we selected the indirect method to extract visual information in our system.

### 2.2. GNSS-Visual Navigation

In 2005, Roberts et al. [24] introduced visual-GNSS navigation and mapping on an aircraft. In their system, the position and velocity of the device are directly measured by the GNSS receiver, while the attitude is calculated by the GNSS and optical flow of the images. Finally, the velocity and optical flow are used to build a depth map of the environment. In [25], the observability of GNSS-aided SfM was studied. The observability of monocular or depth camera-based visual-GNSS navigation systems was analyzed in [12,26]. These studies showed that as long as the system has acceleration that is not parallel to the direction of movement, the scale and the pose of the system are observable. At this point, the theory and practice of visual-GNSS integrated navigation proved to be feasible.

There are different ways to implement visual-GNSS navigation systems. For example, in autonomous driving, pre-built maps for visual localization are often used [10,27,28,29,30,31]. However, such a scheme requires the use of additional maps and can hardly be used in arbitrary scenarios. Additionally, some studies were based on 3 degrees of freedom (DoF) (two-dimensional (2D) position and one-dimensional (1D) direction) localization [10,32,33], whereas in most applications, 6-DoF (three-dimensional (3D) position and attitude) pose estimation is necessary. For universality, our work studied 6-DoF localization without any pre-built maps.

In a tightly coupled visual-GNSS navigation system, the carrier phase or pseudo-range is measured directly and is fused with visual information. In the tightly coupled system, even if only one satellite is tracked, the system can still make use of the GNSS information [34,35]. In a loosely coupled system, the position calculated by the GNSS receiver is fused with visual measurements. At least four satellites must be observed in this scheme. However, in many applications, the user can only achieve the positioning information of the GNSS module, as a loosely coupled system has a wider range of applications. Therefore, we studied loosely coupled visual-GNSS SLAM systems in this paper.

With respect to data fusion, most of the above studies were based on filtering methods. Currently, to the best of our knowledge, only [36] attempted to fuse visual and GNSS data using an iterative optimization-based method with good results. In the field of SLAM, optimization-based algorithms are proven to be more accurate and efficient than filtering-based algorithms. Therefore, our SLAM system is based on optimization.

## 3. System Overview

In GVORB, we need to integrate data from the GNSS receiver and a camera. The system can be divided into two parts: visual-SLAM and data fusion.

**Figure 1:**
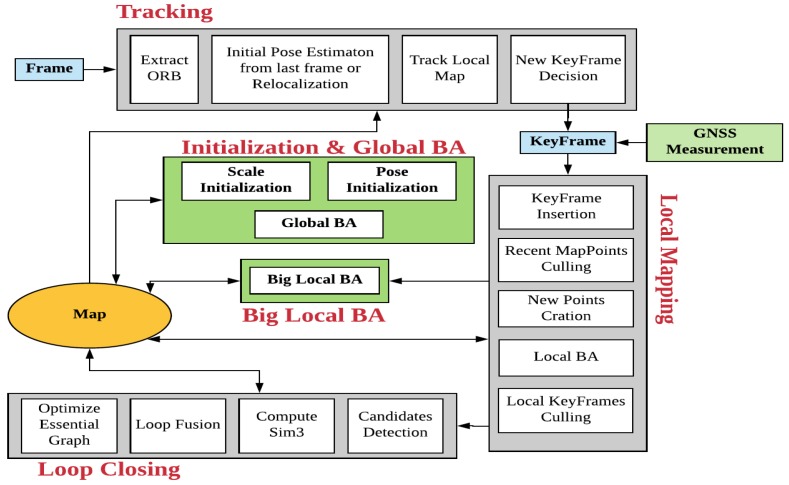
Global Navigation Satellite System–visual-ORB–simultaneous localization and mapping (GVORB) system overview. The gray modules represent the threads of the original ORB-SLAM. The green modules are the new threads in GVORB.

The visual part of GVORB is based on ORB-SLAM2 [37] (https://github.com/raulmur/ORB_SLAM2), an open-source visual-SLAM system. The original ORB-SLAM has three threads: tracking, local mapping, and loop closing (see Figure 1, gray modules). The tracking thread tracks every input frame by matching feature points between the frame and the map and creates keyframes. Then, the local mapping thread builds and refines the local map. Local BA (LBA) is used here to optimize the keyframes and MPs. Finally, the keyframes are sent to the loop closing thread to correct the drift.

For the GNSS and visual information fusion, to improve the performance of a system running on low-cost devices, we present an optimization-based scheme. Two additional threads are added to the system: big LBA (BLBA) and global BA (GBA). The initialization module for aligning GNSS and visual measurements is implemented in the GBA thread (see Figure 1, green modules). The initialization module is divided into two parts. First, if the displacement of the camera is far enough, scale initialization is performed. Then, if the distance moved by the camera is great enough in two orthogonal directions, pose initialization is performed. Afterward, the world and visual coordinates are merged.

After the initialization, BLBA and GBA run separately. GBA optimizes all keyframes poses and MPs with all GNSS and visual observations. The result of GBA is nearly optimal, although it takes a long time. If there are a total of *N* keyframes in the map, the computational complexity is $O(N^{3})$. BLBA is used to integrate GNSS measurements quickly. It only optimizes keyframes and MPs in a local window. However, the local window in BLBA is much bigger than the LBA window in the local mapping thread. This is because in LBA, the local window is too small, and the visual constraints are too strict. GNSS measurements almost do not affect optimization. We did not replace LBA by BLBA because LBA runs faster, which is important for local map updates in fast-motion scenarios. BLBA can be seen as a balance between GBA and LBA. It runs faster than GBA while optimizing more states than LBA.

In GVORB, although the integration of GNSS can avoid SLAM drift, we still run the loop closing thread. This is because loop closure can further improve the system’s consistency. In addition, by finding loop closures, the system understands the real topology of the environment [38]. Further, when a loop is detected, tracking is based on the previously built map, and the map size will not increase when we revisit the same place.

**Figure 2:**
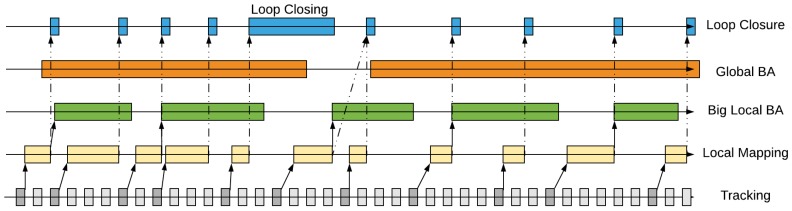
Timing diagram of Global Navigation Satellite System–visual-ORB–simultaneous localization and mapping (GVORB) threads.

The relationship between GVORB threads and their timing diagram is shown in Figure 2:

(1) In the tracking thread, every input frame is tracked. If the frame is selected as a keyframe, it will be sent to the local mapping thread.
(2) The local mapping thread receives the keyframes and then builds and refines the local map. After that, the keyframes are sent to the loop closing thread. At the same time, the BLBA thread is triggered if it is idle.
(3) The BLBA thread optimizes the keyframe poses and MPs in the big local window and then updates the map.
(4) GBA may run at any time but sleeps for a period of time between runs. It optimizes the whole map and updates it.
(5) The loop closing thread runs when it receives the keyframes from the local mapping thread. It detects loops and corrects them. However, in GVORB, this thread does not correct loops, but waits for the GBA thread to do so.

## 4. Modules of GVORB

In the GVORB implementation, we propose GNSS data records, initialization, and the GBA and BLBA modules. We introduce these in this section.

**Figure 3:**
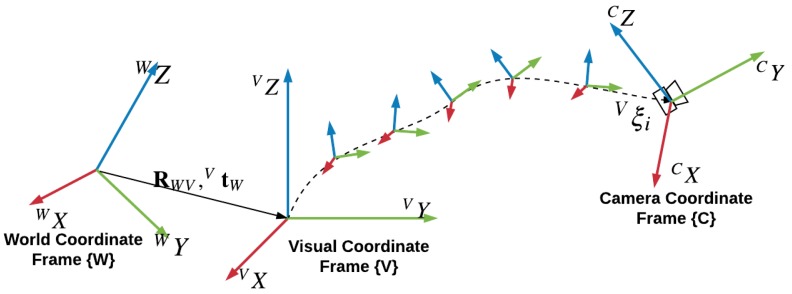
World, visual, and camera coordinate frames.

Three coordinate frames are used in GVORB (see Figure 3). We use the local tangent plane (LTP) as the world coordinate frame, denoted as {W}. GNSS measurements are transformed into the LTP. In visual-SLAM, we place the origin of the visual coordinate frame {V} at the camera’s initial pose. Another frame is the camera coordinate frame, denoted as {C}, which is fixed with the camera. The GNSS receiver is located at $p_{G}$. If the low-cost GNSS receiver is used where the positioning accuracy is not high, we may assume that the GNSS receiver is located at the origin of {C}, that is, $p_{G}={\left[0,0,0,1\right]}^{⊤}$.

### 4.1. GNSS Data Record

**Figure 4:**
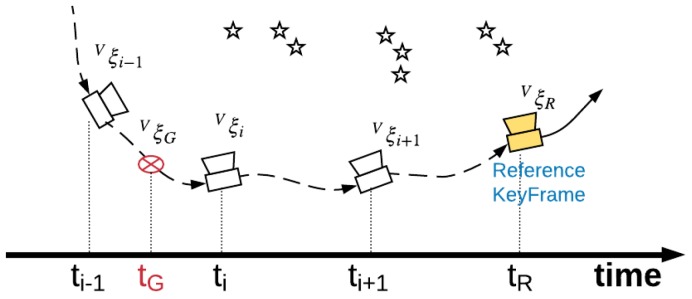
The interpolation of Global Navigation Satellite System (GNSS) positions in a visual navigation system.

Usually, data from two devices is not recorded synchronously. We assume that the timestamp of GNSS data is $t_{G}$, which is between the timestamps of frames i−1 and *i*: $t_{i-1}$ and $t_{i}$. We try to create a keyframe on the basis of the *i*th frame (if the local mapping thread is running, we create the keyframe until it is idle). The new created keyframe works as the reference keyframe, and it is set as non-deletable (to prevent the loop mapping thread from deleting it). The GNSS information is bound to this reference keyframe, as shown in Figure 4.

Because $t_{G}$ is between $t_{i-1}$ and $t_{i}$, the position of the device at $t_{G}$ is between ${\;}^{V}\xi_{i-1}$ and ${\;}^{V}\xi_{i}$ (where ${\;}^{V}\xi_{i}$ represents the pose vector of the camera at $t_{i}$ in {V}). Through first-order interpolation, the device’s pose at $t_{G}$ in {V} can be obtained by:

(1) $${\;}^{V}\xi_{G}={\;}^{V}\xi_{i-1}\circ \frac{t_{G}-t_{i-1}}{t_{i}-t_{i-1}}\left({\;}^{V}\xi_{i-1}^{-1}\circ {\;}^{V}\xi_{i}\right),$$

where ∘ represents the multiplication of two pose vectors: $\xi_{1}\circ \xi_{2}=\log\left(\exp\left(\xi_{1}\right)\exp\left(\xi_{2}\right)\right)$. Then, we convert the pose at $t_{G}$ to the reference keyframe’s coordinate frame {R}, ${\;}^{R}\xi_{G}={\;}^{V}\xi_{G}\circ^{V}\xi_{R}^{-1}$. In {R}, the position of the GNSS receiver at $t_{G}$ is ${\;}^{R}p_{G}=\exp{\left({\;}^{R}\xi_{G}\right)}^{-1}p_{G}$. Afterward, the estimated position of the GNSS receiver in {V} is recorded in the reference keyframe as ${\;}^{R}p_{G}$.

### 4.2. Scale Initialization

The scale of the visual-SLAM system can be initialized when the camera has moved a certain distance and sufficient GNSS measurements have been received. We suppose that in {V}, the estimated position of the GNSS receiver is ${\;}^{V}\hat{P}=\left\{{\;}^{V}{\hat{p}}_{1},{\;}^{V}{\hat{p}}_{2},\ldots ,{\;}^{V}{\hat{p}}_{N}\right\}$ and that the camera trajectory in {W} measured directly by the GNSS receiver is ${\;}^{W}\tilde{P}=\left\{{\;}^{W}{\tilde{p}}_{1},{\;}^{W}{\tilde{p}}_{2},\ldots ,{\;}^{W}{\tilde{p}}_{N}\right\}$, where the standard deviation (STD) of GNSS measurement errors is $\sigma =\{\;\sigma_{1},\sigma_{2},\ldots ,\sigma_{N}\}$. Then, the scale factor ${\hat{s}}_{WV}$ between {W} and {V} can be estimated by

(2) $${\hat{s}}_{WV}=\sqrt{\frac{\sum_{i=1}^{N}{∥{\;}^{W}{\tilde{p}}_{i}-{\;}^{W}\bar{p}∥}^{2}/\sigma_{i}^{2}}{\sum_{i=1}^{N}{∥{\;}^{V}{\hat{p}}_{i}-{\;}^{V}\bar{p}∥}^{2}/\sigma_{i}^{2}}},$$

where ${}^{W}\bar{p}$ and ${}^{V}\bar{p}$ are the mean of the measured and estimated values of the GNSS receivers’ positions, respectively.

After the scale initialization, the entire map is updated with ${\hat{s}}_{WV}$, where the positions of keyframes and MPs are adjusted by ${\hat{s}}_{WV}$. Moreover, the GNSS receivers’ positions in the reference keyframes ${\;}^{R}p_{G}$ are also updated.

### 4.3. Pose Initialization

After scale initialization, the pose between {W} and {V} is aligned. According to [39], the rotation matrix $R_{WV}$ between {W} and {V} can be solved by singular-value decomposition. First, we build a matrix:

(3) $$A=\sum_{i=1}^{N}\left({\;}^{W}{\tilde{p}}_{i}-{\;}^{W}\bar{p}\right)\cdot {\left({\;}^{V}{\hat{p}}_{i}-{\;}^{V}\bar{p}\right)}^{⊤}/\sigma_{i}^{2}.$$

We then perform singular-value decomposition on A: $A=U\Sigma V^{⊤}$. The diagonal elements of Σ are sorted in descending order: $\left[\lambda_{1},\lambda_{2},\lambda_{3}\right]$. If $\lambda_{2}$ is larger than a certain threshold, we start pose initialization. This is because a higher $\lambda_{2}$ leads to higher rotation estimation accuracy. Further, if $\lambda_{3}$ is small, the determinant of U or V may equal −1 (mirror ambiguous). In this case, the last column of U or V should be reversed.

After that, the rotation between the two coordinates can be estimated by $R_{WV}={\mathrm{UV}}^{⊤}$. The relative translation then becomes ${\;}^{V}t_{W}={\;}^{W}\bar{p}-R_{WV}{\;}^{V}\bar{p}$.

After pose initialization, keyframes and MPs in the entire map are transformed by $R_{WV}$ and ${\;}^{V}t_{W}$. Thus, {W} and {V} become coincident. We use {W} to represent them. GBA runs immediately after this to further adjust the visual and GNSS localization results.

### 4.4. Global Bundle Adjustment

**Figure 5:**
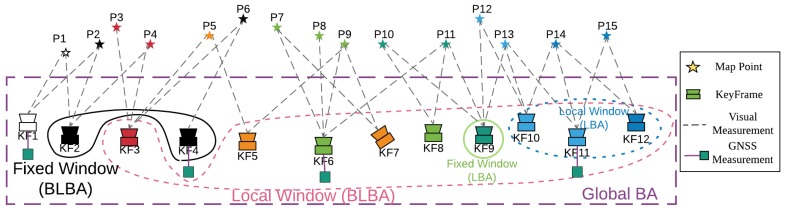
Global Navigation Satellite System–visual-ORB–simultaneous localization and mapping (GVORB) graph optimization model for the local mapping, global bundle adjustment (GBA), and big local BA (BLBA) threads. For simplification, the windows only contain keyframes for each BA.

In the GBA thread, we use all visual and GNSS measurements to constrain the pose of the entire map. The graph model of GBA is shown in Figure 5 (GBA window).

The cost function of Figure 5 can be written as

(4) $$E\left(x\right)=\sum_{i=0}^{N}\sum_{k=0}^{M}E^{vis}\left(i,k\right)+w^{gnss}\sum_{i=0}^{N}E^{gnss}\left(i\right),$$

where x is the state vector to be optimized: $x={\left[\xi_{1}^{⊤},\ldots ,\xi_{N}^{⊤},p_{1}^{⊤},\ldots ,p_{M}^{⊤}\right]}^{⊤}$; $\xi_{i}\dot{=}{\;}^{W}\xi_{i}$ is the *i*th keyframe’s pose; $p_{k}\dot{=}{\;}^{W}p_{k}$ is the *k*th MP’s position; and $w^{gnss}$ is the weight of the GNSS measurements. Because the number of visual measurements is much higher than the number of GNSS measurements, the GNSS measurements need a greater weight to affect the optimization. (Moreover, the GNSS measurement model can be introduced to Equation (4) as a cost term.)

$E^{vis}\left(i,k\right)$ is the cost of the visual measurement of the *i*th frame and *k*th MPs (if not observed, the cost is 0). Here, we use the weighted norm of the reprojection error as a cost function:

(5) $$E^{vis}\left(i,k\right)={∥r^{vis}\left(i,k\right)∥}_{W}^{2}={∥z\left(i,k\right)-\hat{z}\left(i,k\right)∥}_{W}^{2},$$

where ${∥\cdot ∥}_{W}^{2}$ represents the weighted norm: ${∥r^{vis}\left(i,k\right)∥}_{W}^{2}=r^{vis⊤}\left(i,k\right)W_{i,k}^{vis}r^{vis}\left(i,k\right)$; $W_{i,k}^{vis}$ is the information matrix for the measurement; $r^{vis}\left(i,k\right)$ is the residual; z(i,k) is the measurement of the *k*th MP in the *i*th keyframe; and $\hat{z}\left(i,k\right)$ is the predicted value of z(i,k), which is based on the keyframe’s pose estimation ${\hat{\xi }}_{i}$ and MP position’s estimation ${\;}^{W}{\hat{p}}_{k}$:

(6) $$\hat{z}\left(i,k\right)=\pi \left({\;}^{C}{\hat{p}}_{k}\right)=\pi \left(\exp\left({\hat{\xi }}_{i}\right){\;}^{W}{\hat{p}}_{k}\right)=\pi \left({\hat{R}}_{i}{\;}^{W}{\hat{p}}_{k}+{\hat{t}}_{i}\right),$$

where ${\hat{R}}_{i}$ and ${\hat{t}}_{i}$ are the rotation matrix and translation vector for ${\hat{\xi }}_{i}$: $\exp\left({\hat{\xi }}_{i}\right)=\left[\begin{array}{cc} {\hat{R}}_{i} & {\hat{t}}_{i}\\ 0_{3}^{⊤} & 1 \end{array}\right]$; and π is the camera projection function that projects points from 3D space onto the image.

The partial derivative of $r^{vis}\left(i,k\right)$ is

(7) $$\begin{array}{cc} J_{\xi_{i}}^{vis}\left(i,k\right)=\frac{\delta r^{vis}\left(i,k\right)}{\delta {\hat{\xi }}_{i}} & =-\frac{\delta \hat{z}\left(i,k\right)}{\delta {\hat{\xi }}_{i}}=-\frac{\delta \pi ({\;}^{C}{\hat{p}}_{k})}{\delta {\;}^{C}{\hat{p}}_{k}}\cdot \frac{\delta {\;}^{C}{\hat{p}}_{k}}{\delta {\hat{\xi }}_{i}}\\ & =-J^{\pi }\left({\;}^{C}{\hat{p}}_{k}\right)\cdot \left[I_{3}\;-{\left({\hat{R}}_{i}{\;}^{W}{\hat{p}}_{k}+{\hat{t}}_{i}\right)}^{\wedge }\right],\\ J_{p_{k}}^{vis}\left(i,k\right)=\frac{\delta r^{vis}\left(i,k\right)}{\delta {\;}^{W}{\hat{p}}_{k}} & =-J^{\pi }\left({\;}^{C}{\hat{p}}_{k}\right)\cdot {\hat{R}}_{i}. \end{array}$$

Among the terms, $J^{\pi }\left({\;}^{C}{\hat{p}}_{k}\right)$ is the Jacobian matrix of the camera projection function. For the pinhole camera model, this is

(8) $$J^{\pi }\left({\;}^{C}{\hat{p}}_{k}\right)=\frac{1}{{\;}^{C}{\hat{p}}_{zk}}\left[I_{2}\;-\hat{z}\left(i,k\right)\right].$$

Then, the Jacobian matrix of $E^{vis}\left(i,k\right)$ is

(9) $$J^{vis}\left(i,k\right)=\left[0\ldots 0,J_{\xi_{i}}^{vis}\left(i,k\right),0\ldots 0,J_{p_{k}}^{vis}\left(i,k\right),0\ldots 0\right],$$

where the columns of the two non-zero matrix blocks respectively correspond to the rows in which the states $\xi_{i}$ and ${\;}^{W}p_{k}$ are located in x.

The cost of the GNSS measurements is as follows (if there are no GNSS measurements in this keyframe, the cost is 0):

(10) $$E^{gnss}\left(i\right)={∥r^{gnss}\left(i\right)∥}_{\sigma }^{2}={∥{\;}^{W}p_{Gi}-{\;}^{W}{\hat{p}}_{Gi}∥}^{2}/\sigma_{i}^{2},$$

where ${\;}^{W}{\hat{p}}_{Gi}$ is the estimated GNSS position recorded by the *i*th keyframe. It can be calculated from the keyframe’s pose estimation ${\hat{\xi }}_{i}$ and the GNSS position in {R} ${\;}^{R}p_{Gi}$ (where the position is expressed as a homogeneous vector):

(11) $${\;}^{W}{\hat{p}}_{Gi}=\exp\left({\hat{\xi }}_{i}^{-1}\right){\;}^{R}p_{Gi}.$$

The partial derivative of $r^{gnss}\left(i\right)$ is

(12) $$J_{\xi_{i}}^{gnss}\left(i\right)=\frac{\delta r^{gnss}\left(i\right)}{\delta {\hat{\xi }}_{i}}=-\frac{\delta {\;}^{W}{\hat{p}}_{Gi}}{\delta {\hat{\xi }}_{i}}={\hat{R}}_{i}^{-1}\left[I_{3}\;{\left({\;}^{R}p_{Gi}\right)}^{\wedge }\right].$$

The Jacobian matrix of $E^{gnss}\left(i\right)$ then becomes

(13) $$J^{gnss}\left(i,j\right)=\left[0\ldots 0,J_{\xi_{i}}^{gnss}\left(i\right),0\ldots 0\right],$$

where the column of the non-zero matrix block corresponds to the row of the state $\xi_{i}$ in x.

Finally, we combine the residual vectors, Jacobian matrix blocks, and information matrix blocks of all measurements. They are as follows:

(14) $$r=\left[\begin{array}{c} \ldots \\ r^{vis}\left(i,k\right)\\ \ldots \\ r^{gnss}\left(i\right)\\ \ldots \end{array}\right],J=\left[\begin{array}{c} \ldots \\ J^{vis}\left(i,k\right)\\ \ldots \\ J^{gnss}\left(i\right)\\ \ldots \end{array}\right],W=\left[\begin{array}{ccccc} \cdots & & & & \\ & W^{vis}\left(i,k\right) & & & \\ & & \cdots & & \\ & & & W^{gnss}\left(i\right) & \\ & & & & \cdots \end{array}\right]$$

where W only has non-zero matrix blocks on the diagonal, which represents the inverse uncertainty of each measurement, and there is no correlation between the measurements. $W^{vis}\left(i,k\right)=I_{2}/\sigma_{vis,l}^{2}$ is the information matrix for the visual observation; $\sigma_{vis,l}^{2}$ is the location error of the point in the image, which is related to the level *l* of the feature point; and $W^{gnss}\left(i\right)=w^{gnss}\sigma_{i}^{-2}$ is the weight of the GNSS measurement recorded by the *i*th keyframe, which is related to the GNSS positioning error.

The state increment of each iteration of the optimization can be solved by the following equation:

(15) $$J{\left(x\right)}^{⊤}\mathrm{WJ}\left(x\right)\Delta x=-J{\left(x\right)}^{⊤}Wr\left(x\right).$$

After several iterations, all keyframe poses and MP positions in the entire map can be optimized.

### 4.5. Big Local Bundle Adjustment

After the scale initialization, the BLBA thread starts. This thread is similar to the GBA thread, except that BLBA does not optimize the whole map, but instead selects a local window for optimization. However, the local window in BLBA is much larger than that in LBA in the local mapping thread.

In LBA, the local window contains all the covisible keyframes of the current keyframe (keyframes sharing the same MPs are called covisible keyframes) and their visible MPs. Moreover, the keyframes that lie outside the local window but that have covisibility with some keyframes in the window are included in the optimization but remain fixed. For example, in Figure 1, KF12 is the current keyframe, and it observes P14 and P15. Meanwhile, KF10 and KF11 observe the same MP. Thus, KFs 10–12 are in the local window, but the GNSS measurement of KF11 is not used in LBA. Keyframes in the local window observe three MPs: P13–15. KF9 also observes P13. Hence, KF9 is in the fixed window.

However, in BLBA, we set the size of the local window, for example, 8–10 keyframes. We select the keyframes by covisibility iteratively until the number of keyframes in the local window meets the requirements. For example, in Figure 5, KF3 and MPs 5–12 are selected using covisibility several times, and we obtain nine keyframes in the local window. KFs 2 and 4 have covisibility with keyframes in the window and are thus selected by the fixed window. Therefore, the BLBA states are the poses of KF3 and KFs 5–12 and the position of MPs 2–15. Moreover, KF6 and KF11 are constrained not only by visual measurements but also by GNSS measurements.

The cost function of BLBA is similar to that of Equation (4). The only difference is that in BLBA, only states in the local window are optimized. After the optimization, the BLBA thread updates the keyframes and MPs in the local window and waits for the next run.

## 5. Experiments

In this section, we describe how sequences 00–10 of the KITTI odometry dataset [17] were used to evaluate the proposed method. In this dataset, they provide stereo camera and laser scanner data and ground-truth trajectories obtained by the RTK-GPS (Global Positioning System)/IMU (inertial navigation system). The frame rate of the video is about 10 Hz, and the resolution is 1392×512 with a $90^{\circ }\times 35^{\circ }$ field of view. The resolution of the ground-truth data is 0.02 m/0.1${}^{\circ }$. In our experiment, only one camera in the stereo camera system was used. The GNSS measurements were simulated by resampling the ground truth to a 1 Hz trajectory and adding Gaussian white noise with a STD of 3 m by default, which is achievable with a low-cost GNSS. In real practice, the noise model may be more complicated. One may use the pre-processing methods to filter the GNSS measurements [31] or add a cost term in Equation (4) to model the GNSS receiver’s noise.

The tests were performed on an Ubuntu 16.04 system running on an Intel Core i7-4710MQ processor with 16 GB of RAM. The median running time per frame of GVORB was 34 ms, which was slightly slower than that of ORB-SLAM at 32 ms. This was because the tracking threads of the two systems are the same but the GVORB updates the map more frequently; hence it takes slightly more time. In the KITTI dataset, the frame rate was about 10 Hz; hence all the tested systems ran in real-time.

### 5.1. Precision of Initialization

**Figure 6:**
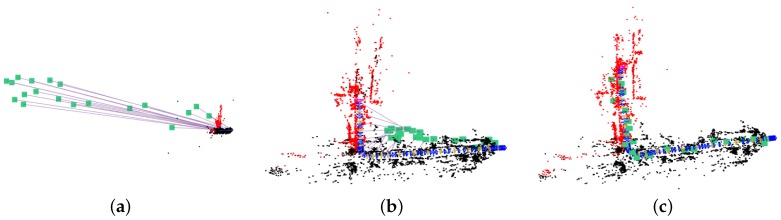
Initialization of Global Navigation Satellite System–visual-ORB–simultaneous localization and mapping (GVORB): (**a**) before initialization; (**b**) after scale initialization; (**c**) after pose initialization. The green square is the location measured by the GNSS receiver, and the blue trajectory comes from visual-SLAM. The purple lines are the connections between GNSS receivers and visual correspondences. The black and red points are the map points (MPs) (the red points are the MPs in the local optimization window).

The GVORB initialization process is shown in Figure 6. At first, the transformation and scale between {W} and {V} were arbitrary. After scale initialization, two coordinates had the same scale but different poses. Finally, after pose initialization, the two coordinates became coherent.

Five initializations were performed on each sequence of the KITTI dataset. When 20 GNSS data points were recorded and the distance moved by the camera was greater than 20 times the GNSS positioning error, initialization was triggered. Table 1 records the scale initialization results.

As shown in the table, when GVORB ran for 20 s, 20 GNSS data points were recorded (in sequence 03, a GNSS measurement was aborted because of the slow motion), and the distance moved exceeded the threshold. Then, the scale initialization started. It can be seen that the scale estimation error was essentially around 3% and that the maximum error was below 6.1%.

After this, if the camera moved a sufficient distance in any two orthogonal directions, pose initialization was triggered. Table 2 shows the results of pose initialization over five runs.

**Figure 7:**
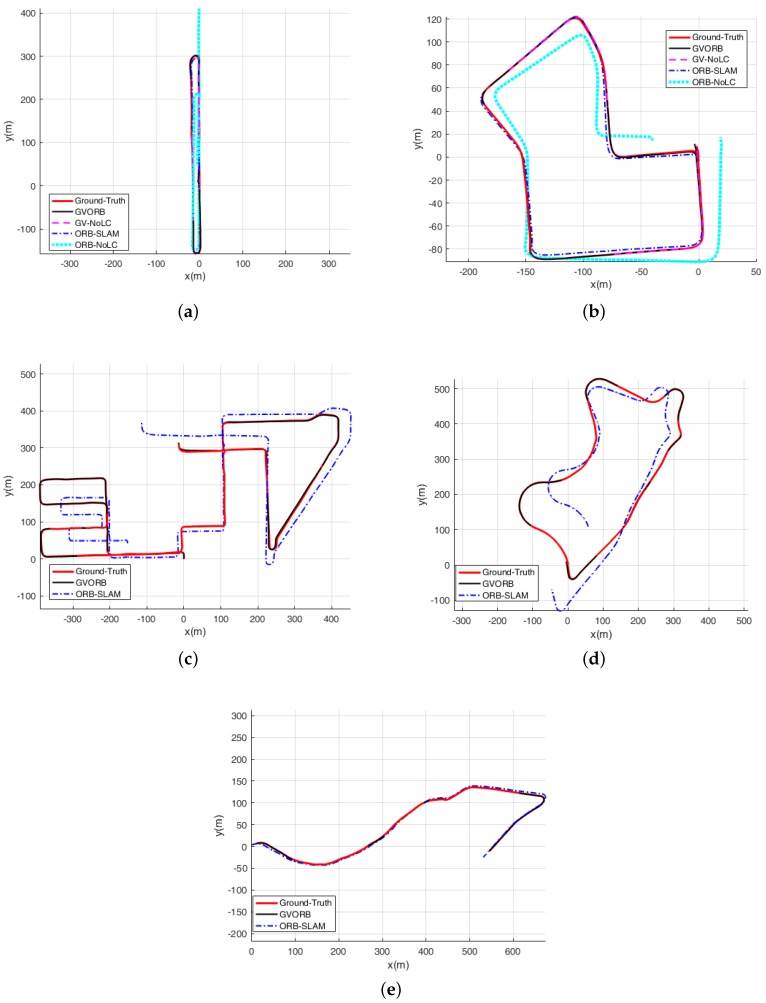
Localization results using the KITTIdataset. blackThe subfigures correspond to sequences 06–10: (**a**) Sequence 06; (**b**) Sequence 07; (**c**) Sequence 08; (**d**) Sequence 09; (**e**) Sequence 10.

In the table, the data for sequences 01 and 04 are missing because the former lost tracking before the pose was initialized (see Section 5.2) and the latter moved straight, which did not meet the initialization criteria. For the other sequences, the initialization times were different depending on the motion conditions. In the successfully initialized sequences, the average rotation error was lower than $4^{\circ }$ and the average position error was lower than 3.6 m. Among the errors, the largest appeared for sequence 06. This was due to the shape of the trajectory being very “narrow” (see Figure 7a). Therefore, the rotation estimation error was relatively large.

### 5.2. Localization Performance

**Figure 8:**
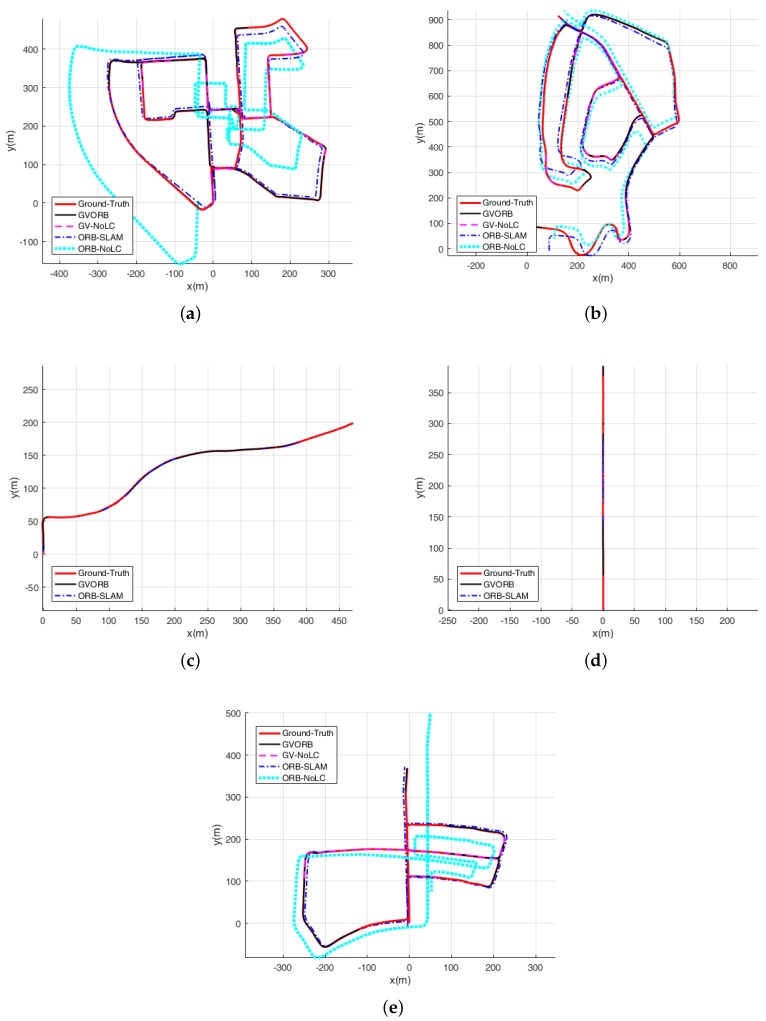
The localization results using the KITTIdataset. The subfigures correspond to sequences 00 and 02–05: (**a**) Sequence 00; (**b**) Sequence 02; (**c**) Sequence 03; (**d**) Sequence 04; (**e**) Sequence 05.

In this section, we describe testing GVORB and ORB-SLAM on the dataset. Considering that in many applications the camera will not visit the same place twice, loop closure is not available, and localization before closing the loop is a good indicator for the accuracy [40]. Therefore, we also tested the no-loop-closure versions of GVORB and ORB-SLAM (abbreviated as GV-NoLC and ORB-NoLC). The localization results are shown in Figure 7 and Figure 8 (blue lines: estimated trajectory; red lines: ground truth). The median root-mean-square error (RMSE) values of position and rotation estimations for all runs are shown in Table 3 and Table 4 (in sequences 03–04 and 08–10, no loops were detected; thus the results of the original and NoLC versions were merged). In sequence 08, although the trajectory overlapped in some regions, the vehicle moved in opposite or orthogonal directions; this is very challenging for place recognition algorithms. Therefore, the systems did not detect any loop in the whole sequence. In sequence 01, all algorithms failed, because this sequence was on a highway with few close trackable objects [1]. Therefore, we do not show the results of this sequence.

From the figures, for all sequences, it can be seen that both GVORB and GV-NoLC worked very well. The estimated and ground-truth trajectories almost overlapped. In Table 3, the median RMSE of the estimated position was 0.82 m, which was much more accurate than measurements taken directly from GNSS receivers (3 m). The accuracy of GV-NoLC was similar to that of GVORB. This is because both visual loop closure and GNSS can correct drift for visual odometry, but in such situations, GNSS performs excellently and loop closure cannot further improve the performance. However, we did not cancel the loop closure thread. The reason is explained in Section 3.

The algorithms developed in [27,30] integrate electronic maps and low-cost GNSS and IMUs. When the GNSS receiver’s accuracy is also 3 m, the positioning error is higher than 0.7 m. Our GVORB system achieves similar accuracy without any pre-built map or IMU.

For sequences 03, 04, and 07, the accuracies of ORB-SLAM and GVORB were similar. These three sequences are relatively short, and visual error accumulation was not significant. For sequences 00 and 05, although they are long, loop closure was performed several times, and thus the error was not high. Although there was a loop correction for sequence 02, it was not enough to effectively constrain all the trajectories. Sequences 08 and 09 are very long, but the system did not detect any loops. Thus, the positioning errors of sequences 02, 08, and 09 were much higher than for GVORB. Further, ORB-NoLC does not perform loop closure; thus, as a result of error accumulation, the positioning error was very high for all long sequences.

From Table 4, the attitude accuracies estimated by every method were close for all sequences. With the exception of sequence 10, the median RMSE values were around $1^{\circ }$. GVORB was only slightly better than ORB-SLAM, and the loop closure slightly raised the attitude error. The results show that for the KITTI dataset, the attitude drift of visual-SLAM was very small.

The above results show that pure monocular visual-SLAM only guarantees positioning accuracy within a certain time and distance range. In the experiment, the positioning error was less than 5 m for 2 min and 1 km. When integrated with GNSS measurements, GVORB achieved very high positioning accuracy over any long trajectory.

### 5.3. GNSS Noise versus Localization Error

**Figure 9:**
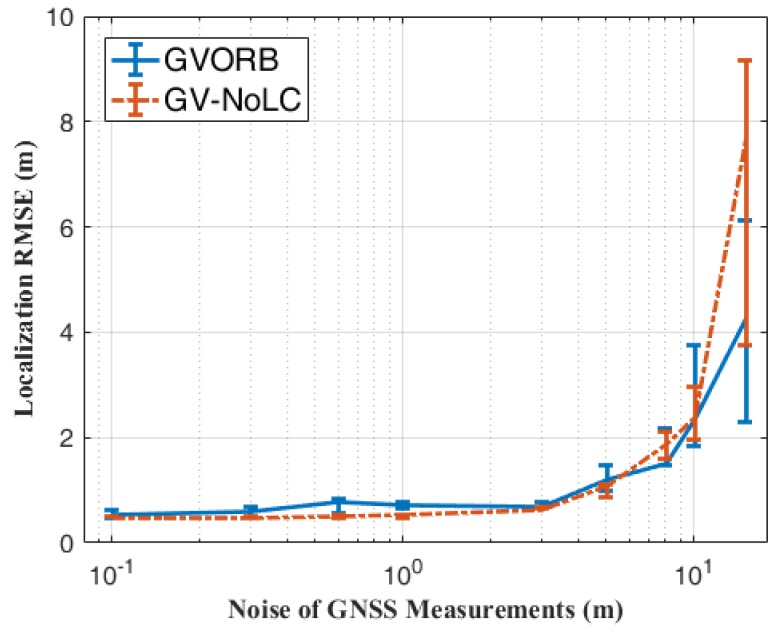
Localization root-mean-square error (RMSE) of Global Navigation Satellite System–visual- ORB–simultaneous localization and mapping (GVORB) and no-loop-closure version (GV-NoLC) with respect to GNSS noise.

We tested the effect of the GNSS error on the localization error of GVORB and GV-NoLC for sequence 05. Noise was added to the ground-truth trajectory to simulate noisy GNSS data. The STDs of the GNSS noise were set to [0.1,0.3,0.6,1,3,5,8,10,15] m. The localization errors are shown in Figure 9.

From the figure, when the GNSS noise was less than 1 m, the localization error of GVORB was always around 0.6 m. In this situation, the GVORB error was higher than that of GNSS. This was mainly due to the fact that the GNSS frame rate was 1 Hz and the frame rate of the camera was about 10 Hz. Thus, only 1/10 of the frames could be constrained by the GNSS receivers. The visual error of the other frames played a major role in the localization error. Therefore, the overall error was slightly higher than the GNSS noise. When the GNSS noise increased from 1 to 15 m, although the GVORB error also increased, the growth rate was relatively low; it only increased from 0.7 to 4 m. The error was about 1/4 of the GNSS noise. In this situation, the weights of the GNSS measurements were decreased automatically; hence the error grew sublinearly with GNSS noise.

GVORB and GV-NoLC demonstrated similar performances when the GNSS noise was less than 5 m. We can conclude that in this case, GNSS and loop closure had similar abilities to reduce drift. When the GNSS noise grew larger, loop closure played a more important role in reducing the localization error. Comparing GV-NoLC with ORB-NoLC, for sequence 05, the former achieved 8 m accuracy in the worst case, while the error of the latter reached 26 m. We can conclude that when there is no loop in the trajectory, GVORB greatly outperforms pure GNSS and visual-SLAM.

## 6. Discussion

In the previous section, we evaluate GVORB in terms of the KITTI dataset. Experiments showed that GVORB incorporates the benefits of GNSS and visual-SLAM. The system estimates absolute pose with high accuracy and scale with no drift.

In addition, the original ORB-SLAM method has the ability to relocalize from a pre-built map. Although we did not test it, GVORB inherits this ability. Further, the map built by GVORB can be also utilized by pure visual-SLAM systems. GVORB can also be integrated with the other latest SLAM technologies. For example, the method in this article can be extended to stereo, panorama, and RGB-D cameras [37] or can be combined with IMU [41] to further improve the accuracy and robustness of the system. In addition, the map in GVORB is very sparse, which is only useful for localization. It is also possible and easy to add semi-dense [42] and dense [6,43] mapping modules. By combining the scene understanding module, GVORB can also work for obstacle avoidance [5] and scene segmentation and understanding [6,7,44].

## 7. Conclusions

In this paper, we propose GVORB: an integrated visual-GNSS–SLAM system. In the system, a monocular camera and low-cost GNSS with a positioning accuracy of several meters were applied and studied. Compared with pure GNSS, GVORB can measure attitude, and it has higher localization accuracy. Compared to the visual-SLAM system, absolute pose and scale are observable in GVORB and drift is eliminated, which greatly improves the long-term localization accuracy. Experiments on the KITTI dataset verified the above conclusion. Through the experiments, we found that GNSS measurements could provide higher consistency compared to visual loop closure. Loop closure does not significantly improve the localization accuracy of GVORB when the GNSS positioning error is relatively small (less than 10 m). Benefitting from the smooth effect of visual-SLAM, GVORB obtains a higher frame rate and accuracy than pure GNSS positioning. In the case of poor GNSS accuracy, the performance of GVORB is much better than that of pure GNSS positioning.

In addition, unlike other visual-GNSS navigation systems, we use an optimization-based method for data fusion. Therefore, our method has high accuracy. Our method demonstrated a performance similar to that of navigation algorithms that integrate cameras, IMUs, GNSS receivers, and pre-built maps. Moreover, our method also creates a global map that has global consistency during long-term navigation.

Furthermore, our system can be extended to work on other types of cameras and to build other types of maps. This is a direction we will study in the future.

## Figures and Tables

**Table 1:** Scale initialization of Global Navigation Satellite System–visual-ORB–simultaneous localization and mapping (GVORB).

Sequence	00	01	02	03	04	05	06	07	08	09	10
**Triggering Time (s)**	20	20	20	21	20	20	20	20	20	20	20
**Trajectory Length (m)**	142	413	224	132	275	150	230	119	164	186	168
**Scale Error (%)**											
Mean	3.6	4.7	2.5	2.3	1.2	3.0	4.1	3.7	2.5	2.5	1.1
Median	3.9	4.5	2.8	2.2	1.3	2.5	3.5	3.5	2.6	1.9	0.3
Max	4.0	5.3	4.1	2.5	1.8	4.3	3.5	4.8	4.9	6.1	3.8

**Table 2:** Pose initialization of Global Navigation Satellite System–visual-ORB–simultaneous localization and mapping (GVORB).

Sequence	00	01	02	03	04	05	06	07	08	09	10
**Triggering Time (s)**	34	—	22	51	—	24	33	23	42	25	26
**Trajectory Length (m)**	244	—	253	373	—	177	322	140	406	238	210
**Rotation Error (${}^{\circ }$)**											
Mean	1.6	—	1.5	1.2	—	1.1	4.8	1.5	4.1	2.5	2.4
Median	1.4	—	1.5	1.0	—	1.0	2.7	1.7	3.8	2.3	3.0
Max	2.3	—	3.4	1.9	—	1.6	12.3	2.1	7.2	4.6	3.0
**Position Error (m)**											
Mean	2.1	—	2.1	2.1	—	3.1	3.1	1.9	2.6	2.4	3.7
Median	2.2	—	2.2	1.9	—	2.1	3.2	1.0	2.6	2.5	3.6
Max	2.3	—	3.3	2.8	—	6.6	3.3	5.0	5.4	4.0	4.0

**Table 3:** Median root-mean-square error (RMSE) values of position estimation (m).

Sequence	GVORB	GV-NoLC	ORB-SLAM	ORB-NoLC
00	0.64	0.94	5.45	45.00
02	1.02	0.97	17.29	24.12
03	0.64		0.62	
04	0.63		0.68	
05	0.71	0.65	3.11	26.24
06	1.13	0.88	6.85	31.62
07	0.66	0.66	1.17	11.01
08	1.26		28.20	
09	0.74		31.46	
10	0.69		3.92	

**Table 4:** Median root-mean-square error (RMSE) values of rotation estimation (${}^{\circ }$).

Sequence	GVORB	GV-NoLC	ORB-SLAM	ORB-NoLC
00	0.83	1.11	1.26	1.21
02	0.83	0.73	1.23	1.18
03	0.57		0.44	
04	0.27		0.12	
05	0.59	0.61	0.69	0.66
06	1.36	1.33	0.80	0.82
07	0.74	0.74	0.96	0.74
08	0.96		1.18	
09	0.64		1.05	
10	0.54		4.11	

## Abbreviations

The following abbreviations are used in this manuscript:

SLAM	Simultaneous localization and mapping
VO	Visual odometry
GNSS	Global Navigation Satellite System
BA	Bundle adjustment
MP	Map point
STD	Standard deviation
DoF	Degrees of freedom
RMSE	Root-mean-square error

## References

[1] Mur-Artal R., Montiel J.M.M., Tardos J.D. (2015). ORB-SLAM: A versatile and accurate monocular SLAM system. IEEE Trans. Robot..

[2] Engel J., Koltun V., Cremers D. (2018). Direct sparse odometry. IEEE Trans. Pattern Anal. Mach. Intell..

[3] Leutenegger S., Lynen S., Bosse M., Siegwart R., Furgale P. (2015). Keyframe-based visual—Inertial odometry using nonlinear optimization. Int. J. Robot. Res..

[4] Liu Y., Chen Z., Zheng W., Wang H., Liu J. (2017). Monocular Visual-Inertial SLAM: Continuous Preintegration and Reliable Initialization. Sensors.

[5] Ling Y., Shen S. Building maps for autonomous navigation using sparse visual SLAM features. Proceedings of the 2017 IEEE/RSJ International Conference on Intelligent Robots and Systems (IROS).

[6] Tateno K., Tombari F., Laina I., Navab N. (2017). CNN-SLAM: Real-time dense monocular SLAM with learned depth prediction. arXiv.

[7] Tateno K., Tombari F., Navab N. When 2.5 D is not enough: Simultaneous reconstruction, segmentation and recognition on dense SLAM. Proceedings of the 2016 IEEE International Conference on Robotics and Automation (ICRA).

[8] Knuth J., Barooah P. (2013). Error growth in position estimation from noisy relative pose measurements. Robot. Auton. Syst..

[9] Huang S., Dissanayake G. (2016). A critique of current developments in simultaneous localization and mapping. Int. J. Adv. Robot. Syst..

[10] Levinson J., Thrun S. Robust vehicle localization in urban environments using probabilistic maps. Proceedings of the 2010 IEEE International Conference on Robotics and Automation (ICRA).

[11] Agrawal M., Konolige K. Real-time localization in outdoor environments using stereo vision and inexpensive GPS. Proceedings of the ICPR 2006 18th International Conference on Pattern Recognition.

[12] Dusha D., Mejias L. (2012). Error analysis and attitude observability of a monocular GPS/visual odometry integrated navigation filter. Int. J. Robot. Res..

[13] De Ponte Müller F. (2017). Survey on ranging sensors and cooperative techniques for relative positioning of vehicles. Sensors.

[14] Bonin-Font F., Ortiz A., Oliver G. (2008). Visual navigation for mobile robots: A survey. J. Intell. Robot. Syst..

[15] Chao H., Cao Y., Chen Y. (2010). Autopilots for small unmanned aerial vehicles: A survey. Int. J. Control Autom. Syst..

[16] Rublee E., Rabaud V., Konolige K., Bradski G. ORB: An efficient alternative to SIFT or SURF. Proceedings of the 2011 IEEE international conference on Computer Vision (ICCV).

[17] Geiger A., Lenz P., Urtasun R. Are we ready for autonomous driving? the KITTI vision benchmark suite. Proceedings of the 2012 IEEE Conference on Computer Vision and Pattern Recognition (CVPR).

[18] Smith R.C., Cheeseman P. (1986). On the representation and estimation of spatial uncertainty. Int. J. Robot. Res..

[19] Montemerlo M., Thrun S., Koller D., Wegbreit B. FastSLAM: A factored solution to the simultaneous localization and mapping problem. Proceedings of the AAAI/IAAI National Conference on Artificial Intelligence.

[20] Davison A.J. Real-time simultaneous localisation and mapping with a single camera. Proceedings of the Ninth IEEE International Conference on Computer Vision.

[21] Klein G., Murray D. Parallel tracking and mapping for small AR workspaces. Proceedings of the ISMAR 2007 6th IEEE and ACM International Symposium on Mixed and Augmented Reality.

[22] Strasdat H., Montiel J., Davison A.J. Real-time monocular SLAM: Why filter?. Proceedings of the 2010 IEEE International Conference on Robotics and Automation (ICRA).

[23] Engel J., Schöps T., Cremers D. (2014). LSD-SLAM: Large-scale direct monocular SLAM. Computer Vision: Proceedings of the ECCV 2014 13th European Conference, Zurich, Switzerland, 6–12 September 2014.

[24] Roberts P., Walker R., O’Shea P. Fixed wing UAV navigation and control through integrated GNSS and vision. Proceedings of the AIAA Guidance, Navigation, and Control Conference and Exhibit.

[25] Carceroni R., Kumar A., Daniilidis K. Structure from motion with known camera positions. Proceedings of the 2006 IEEE Computer Society Conference on Computer Vision and Pattern Recognition.

[26] Dusha D., Mejias L., Walker R. (2011). Fixed-wing attitude estimation using temporal tracking of the horizon and optical flow. J. Field Robot..

[27] Shunsuke K., Yanlei G., Hsu L.T. GNSS/INS/on-board camera integration for vehicle self-localization in urban canyon. Proceedings of the 2015 IEEE 18th International Conference on Intelligent Transportation Systems (ITSC).

[28] Parra I., Sotelo M.A., Llorca D.F., Fernández C., Llamazares A., Hernández N., García I. Visual odometry and map fusion for GPS navigation assistance. Proceedings of the 2011 IEEE International Symposium on Industrial Electronics (ISIE).

[29] Tao Z., Bonnifait P. Tightly coupling GPS with lane markings for autonomous vehicle navigation. Proceedings of the 2014 IEEE 17th International Conference on Intelligent Transportation Systems (ITSC).

[30] Suhr J.K., Jang J., Min D., Jung H.G. (2017). Sensor fusion-based low-cost vehicle localization system for complex urban environments. IEEE Trans. Intell. Transp. Syst..

[31] Tao Z., Bonnifait P., Frémont V., Ibanez-Guzman J., Bonnet S. (2017). Road-Centered Map-Aided Localization for Driverless Cars Using Single-Frequency GNSS Receivers. J. Field Robot..

[32] Senlet T., Elgammal A. A framework for global vehicle localization using stereo images and satellite and road maps. Proceedings of the 2011 IEEE International Conference on Computer Vision Workshops (ICCV Workshops).

[33] Schleicher D., Bergasa L.M., Ocaña M., Barea R., López M.E. (2009). Real-time hierarchical outdoor SLAM based on stereovision and GPS fusion. IEEE Trans. Intell. Transp. Syst..

[34] Soloviev A., Venable D. Integration of GPS and vision measurements for navigation in GPS challenged environments. Proceedings of the 2010 IEEE/ION Position Location and Navigation Symposium (PLANS).

[35] Gakne P.V., O’Keefe K. (2018). Tightly-Coupled GNSS/Vision Using a Sky-Pointing Camera for Vehicle Navigation in Urban Areas. Sensors.

[36] Shi Y., Ji S., Shi Z., Duan Y., Shibasaki R. (2013). GPS-supported visual SLAM with a rigorous sensor model for a panoramic camera in outdoor environments. Sensors.

[37] Mur-Artal R., Tardós J.D. (2017). ORB-SLAM2: An open-source SLAM system for monocular, stereo, and RGB-D cameras. IEEE Trans. Robot..

[38] Cadena C., Carlone L., Carrillo H., Latif Y., Scaramuzza D., Neira J., Reid I., Leonard J.J. (2016). Past, present, and future of simultaneous localization and mapping: Toward the robust-perception age. IEEE Trans. Robot..

[39] Arun K.S., Huang T.S., Blostein S.D. (1987). Least-squares fitting of two 3-D point sets. IEEE TranS. Pattern Anal. Mach. Intell..

[40] Engel J., Usenko V., Cremers D. (2016). A photometrically calibrated benchmark for monocular visual odometry. arXiv.

[41] Mur-Artal R., Tardós J.D. (2017). Visual-inertial monocular SLAM with map reuse. IEEE Robot. Autom. Lett..

[42] Mur-Artal R., Tardós J.D. Probabilistic Semi-Dense Mapping from Highly Accurate Feature-Based Monocular SLAM. Proceedings of the Robotics: Science and Systems.

[43] Yang Z., Gao F., Shen S. Real-time monocular dense mapping on aerial robots using visual-inertial fusion. Proceedings of the 2017 IEEE International Conference on Robotics and Automation (ICRA).

[44] Salas-Moreno R.F., Newcombe R.A., Strasdat H., Kelly P.H., Davison A.J. Slam++: Simultaneous localisation and mapping at the level of objects. Proceedings of the 2013 IEEE Conference on Computer Vision and Pattern Recognition (CVPR).

